# Structural and Biological Characterizations of Novel High-Affinity Fluorescent Probes with Overlapped and Distinctive Binding Regions on CXCR4

**DOI:** 10.3390/molecules24162928

**Published:** 2019-08-13

**Authors:** Siyu Zhu, Qian Meng, Robert T. Schooley, Jing An, Yan Xu, Ziwei Huang

**Affiliations:** 1 School of Life Sciences, Tsinghua University, Beijing 100084, China; 2 Department of Medicine, Division of Infectious Diseases and Global Public Health, School of Medicine, University of California San Diego, La Jolla, CA 92037, USA; 3 School of Life and Health Sciences, Chinese University of Hong Kong, Shenzhen 518172, China

**Keywords:** CXC-type chemokine receptor 4 (CXCR4), HIV-1 infection, cancer metastasis, fluorescent peptidic probes, distinct subpockets, competitive ligand binding, molecular docking simulations

## Abstract

CXC-type chemokine receptor 4 (CXCR4) is well known as a co-receptor for cellular entry and infection of human immunodeficiency virus type 1 (HIV-1). As an important member of the G protein-coupled receptor (GPCR) family, CXCR4 also mediates a variety of cellular processes and functions, such as cell chemotaxis, proliferation, and calcium signal transductions. Identification and characterization of molecular ligands or probes of CXCR4 have been an intensive area of investigations as such ligands or probes are of significant clinical values for the studies and treatments of HIV-1 infection and other human diseases mediated by the receptor. The crystal structures of CXCR4 in complex with different ligands have revealed two distinctive binding regions or subpockets. Thus, understanding the interactions of diverse ligands with these distinctive CXCR4 binding regions has become vital for elucidating the relationship between binding modes and biological mechanisms of ligand actions. Peptidic CVX15 is the only ligand that has been validated to bind one of these distinctive binding regions (or so called the major subpocket) of CXCR4. Therefore, in this study, we developed an efficient probe system including two high-affinity peptidic fluorescent probes, designated as FITC-CVX15 and FITC-DV1, with the aim of targeting distinctive CXCR4 subpockets. We conducted rational design and chemical characterization of the two CXCR4-specific probes and examined their application in biological experiments including competitive binding assays, flow cytometry analysis, and confocal imaging. Especially these two probes were applied in parallel CXCR4 competitive binding assays to detect and analyze potential binding modes of diverse CXCR4 ligands, together with molecular docking and simulations. Our results have indicated that these peptidic fluorescent probe systems provide novel ligand detecting tools, as well as present a new approach for analyzing distinctive binding modes of diverse CXCR4 ligands.

## 1. Introduction

**Chemokine receptor CXCR4 and therapeutic significance.** CXCR4 is a G protein-coupled receptor possessing seven-transmembrane helices that interacts with its endogenous ligand, CXCL12 (SDF-1α) [1]. The resulting CXCR4-SDF-1α biological axis regulates multiple cellular signaling pathways that are associated with cell proliferation, migration, and inflammation. In addition to the natural SDF-1α, various synthetic CXCR4 ligands have been developed [2]. CXCR4 plays a critical role in numerous physiological and pathological processes, including cellular behaviors such as proliferation and chemotaxis, as well as downstream intracellular signaling, such as calcium flux and gene transcriptions [3,4]. Therefore, it is a promising therapeutic target protein for HIV-1 infection, hematological disorders, and metastatic tumors [3,5,6,7,8,9]. The interactions of CXCR4 receptor with diverse ligands, including small molecules and peptides, modulate downstream biochemical signaling involving PI3 kinase pathway, Janus kinase (JAK)/signal transducer, and activator of transcription (STAT) pathway [2] and therefore have potential clinical applications for treating metastatic tumors and AIDS.

**CXCR4 peptide and peptidomimetic ligands.** Peptide and peptidomimetic ligands of CXCR4 are derivatives of antimicrobial peptides, endogenous ligands SDF-1α or viral chemokine-like protein vMIP-II sequence [10]. Discovery of peptidic inhibitors targeting CXCR4 originated from two peptides, tachyplesin and polyphemusin, firstly extracted from hemocytes of horseshoe crabs. These peptides were subsequently optimized into well-known peptides T22, T140, and a series of related analogues [11,12,13,14,15]. Further research with the combination of molecular size reduction approach and compound library screening developed various efficient cyclic peptides including FC131 and POL3026 [16,17]. In addition, arginine-abundant peptides such as ALX40-4C were further proven to directly target CXCR4 and effectively block HIV-1 entry [18]. Multiple truncations and analogues of SDF-1α and vMIP-II with high CXCR4 binding affinity and anti-cancer or anti-HIV potency were also identified recently years based on rational design or biological screening [19,20,21,22].

High-affinity peptides (HAPs) are normally derived from naturally occurring protein-protein interactions (PPIs) and further optimized with unnatural amino acids or peptidomimetics [23]. They are typically small in molecular weight and nontoxic, but usually show more specific binding to their target proteins with higher affinities. For these reasons, HAPs are widely engineered for specific biotechnological applications including imaging probes and clinical therapeutic tools. CXCR4-targeting HAPs are generally derived from cyclic peptide CVX15 series [24] and natural chemokine vMIP-II series [25]. Crystal structures of these HAPs in complex with CXCR4 have revealed a classic two-subpocket binding model [25]. CVX15 was proven to locate in the major pocket of CXCR4 by closely interacting with residue Asp262 [24], whereas N-terminus of vMIP-II in the crystal complex structure reached into the minor pocket of CXCR4 by interacting with residues Trp94, Asp97 and Glu288 [25]. These two distinct binding sites led to insightful biological and computational analysis for diverse ligands that might adopt different binding poses.

**Molecular probes for CXCR4 imaging and detection.** Fluorescent and isotope labeling are conventional approaches for qualitative or quantitative detection of ligand-receptor interactions. Importance of CXCR4 in clinical detection and therapeutics makes the development of imaging agents for CXCR4 highly desirable over the past decades [26,27,28,29,30]. Peptide agonists and antagonists including SDF-1α and T140 peptides can be readily labeled with isotopes, thereby enabling visualization studies of CXCR4 inside tumors. Well-known direct radio-labeling for CXCR4 ligands includes [^125^I]SDF-1α [26], [^99^mTc]SDF-1α [31], (^64^)Cu-T140-2D [32], 4-[^18^F]-T140 [33] and [^18^F]RPS-544 [34]. Alternatively, imaging probes such as [^111^In]-DTPA-Ac-TZ14011 [35], [^64^Cu]-DOTA-NFB, and [^64^Cu]-NOTA-NFB utilize CXCR4 ligands as a carrier for delivery [29]. Anti-CXCR4 antibodies such as 12G5 can also be radiolabeled as [^125^I]-12G5 for clinical immuno-imaging of CXCR4 [36]. In addition, small molecule-based probes including [^64^Cu]-Plerixafor [28], [^99^mTc]O_2_-Plerixafor [37], [^11^C]methyl-AMD3465 [38] and [^68^Ga]-Pentixafor [39,40] were developed as a hybrid molecule or coupled with linkers like PEG or with chelators [30]. Practical safety problems of radioactive probes impelled the development of nonradioactive fluorescence-labeled CXCR4 probes. Fluorescein isothiocyanate-conjugated SDF-1α (FITC-SDF-1α) was synthesized to trace internalization of CXCR4 on endothelial cells [41], and AF647-SDF-1α [42] was presented in previous studies as an alternative to radiolabeled SDF-1α for the evaluation of CXCR4 expression levels. Other fluorescent probes included FAM-DV1 [22] and Ac-TZ14011 labeled with FITC [43], or TAMRA [44], as well as other type of fluorophores [45]. FAM-DV1 was developed by coupling fluorescein to the NH2 group of the side chain of residue Lys17 on DV1 sequence. Moreover, biotin-labeled molecular probes for CXCR4 with biotin-TN14003 as a representative [46] were also used in binding assays for initial screening of anti-CXCR4 small molecules by microscopy. Biotin-Acp-TZ14011 exhibited specific and high affinity for CXCR4 as well [45]. Combination of [^125^I]SDF-1α and fluorescence-labeled antibodies, either covalently attached with phycoerythrin (PE) [47] or coupled with FITC-conjugated goat anti-mouse IgG [15,22], has led to the development of a number of CXCR4 binding assays in recent years. However, isotope-labeled binding assays are radioactive and usually have poor stability and high background signals, and the 12G5-based binding assays are costly and time-consuming. Therefore, developing simpler, nonradioactive and more specific probes for CXCR4 binding detection is of great significance.

Our group has a long-term interest in both understanding precise and distinguished binding modes of diverse CXCR4 ligands as well as developing novel molecular probes. ‘Two distinct subpockets’ binding model opened a new chapter for ligand binding mode analysis of CXCR4 and CVX15 is for now the only ligand proven by crystallography to locate in the major pocket. The above information in combination brought us a new perspective for analyzing CXCR4-ligand binding details. Here in this study, we developed an efficient probe system including two high-affinity peptidic fluorescent probes, designated as FITC-CVX15 and FITC-DV1, with the aim of targeting distinct CXCR4 subpockets. We characterized FITC-CVX15 and FITC-DV1 [48] and subsequently applied them to a cell-based competitive binding system including three parallel binding assays. Biochemical and computational approaches were also combined to elucidate binding affinities and interactive patterns between CXCR4 and diverse ligands.

## 2. Results

### 2.1. Synthesis and Characterization of New Peptidic CXCR4 Probes

**Figure 1:**
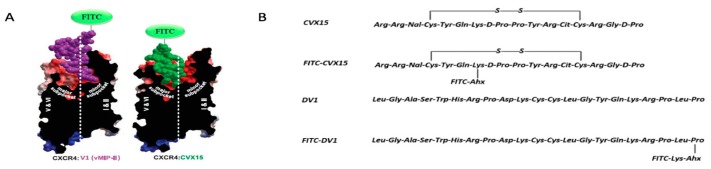
(**A**) Schematic diagrams of FITC-CVX15 and FITC-DV1 in complex with CXCR4 based on co-crystal structures. (**B**) Sequences and schematic structures of CVX15, FITC-CVX15, DV1 and FITC-DV1.

CXCR4-targeting HAP CVX15 was primarily derived from a natural β-hairpin peptide polyphemusin-II [11] and subsequently truncated peptide POL3026 [17]. It is a 16-amino acid-containing cyclic peptide with a disulfide linkage and shows higher affinity for CXCR4 among all polyphemusin-II-derived CXCR4 antagonists. DV1 peptide [22] is an all d-amino acid analog of V1 peptide [21], which was initially derived from N-terminus of natural ligand protein vMIP-II. As we previously reported, DV1 unexpectedly displayed stronger binding and antagonistic activity toward CXCR4 compared to its l-amino acid analog V1. Site-directed mutagenesis studies were performed in our laboratory to identify critical interactive residues for V1 and DV1 binding to CXCR4. Together with co-crystal structure of chemokine vMIP-II, we predicated that N-terminus of DV1 peptide recognizes minor pocket of CXCR4 whereas CVX15 interacts closely with major pocket. We synthesized two high-affinity fluorescent probes by introducing FITC into CVX15 and DV1 (Figure 1A). Our chemical design was based on crystal structures of CVX15-CXCR4 [24] and vMIP-II-CXCR4 [25] in order to maintain their native interactions with CXCR4 while introducing fluorescent fragments to original peptide structures. CVX15 was labeled with a flexible linkage on residue Lys7 which is theoretically located outward from the major pocket (Figure 1B). FITC-DV1 was synthesized by conjugating FITC to the C-terminus of DV1 peptide, as was previously reported by our laboratory [48]. They were further characterized by Matrix-assisted laser desorption ionization time-of-flight (MALDI-TOF) mass spectrometry analysis and High-Performance Liquid Chromatography (HPLC).

**Figure 2:**
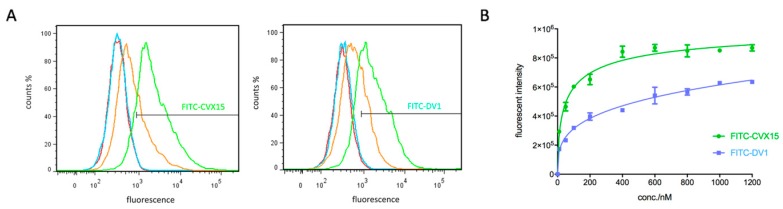
(**A**) FITC-CVX15 and FITC-DV1-labeled CHO-CXCR4 cell lines detected by FCM respectively. Red, blue, orange and green signals represent concentrations of 1 nM, 10 nM, 100 nM and 1000 nM respectively. (**B**) Saturation curves for binding of FITC-CVX15 and FITC-DV1 to CXCR4. Mean ± standard deviation; *n* = 3 independent experiments.

Binding affinity and sensitivity of FITC-CVX15 and FITC-DV1 probes were then evaluated by in vitro CXCR4-overexpressing cell lines (Figure 2A). Flow cytometry (FCM) studies indicated these labeled peptide probes recognized CXCR4 selectively in a concentration dependent manner. Binding saturation curves showed that FITC-CVX15 presented higher affinity than FITC-DV1 to CXCR4 as we expected (Figure 2B). Fluorescent signals of FITC-CVX15 reached a plateau at a concentration of 400 nM whereas the signal of FITC-DV1 was plateaued at 1 μM, which was in agreement with our previously published data [48]. Biological data and molecular modeling studies also indicated that fluorescent labels introduced no destructive effect on the original structures of CVX15 and DV1 and did not affect their binding to CXCR4. Besides, we tested the CXCR4 selectivity of FITC-CVX15 and FITC-DV1 on CCR5-overexpressing CHO cell lines under a concentration of 5 μM that was much higher than their saturation concentrations (Figure S1). Both of them showed none specific binding on CCR5 overexpression, indicating these two probes have no cross-reactivity with CCR5.

### 2.2. Applications in CXCR4-Ligand Competitive Binding Assays

Subsequently, this FITC-labeled probe system was utilized in CXCR4 competitive binding assays with 400 nM of FITC-CVX15 and 1 μM of FITC-DV1 as their saturation concentrations, and antibody 12G5 at a concentration of 250 ng/mL. FITC-CVX15-based, FITC-DV1-based, and 12G5-based competitive binding assays were then conducted in parallel for ten classic CXCR4 ligands as representatives, including both small organic molecules and polypeptides (Table 1). All these molecules showed competitive inhibitory effects with labeled probes. IC_50_ values for each ligand were calculated by regression analysis and each compound presented IC_50_ values within the same order of magnitude from three parallel assays. Small organic molecules, such as IT1t, AMD070, and unnatural polypeptides, including CVX15 and HC4319 showed relatively high binding affinities to CXCR4 with IC_50_ values of less than 50 nM. Our results confirmed that FITC-CVX15 and FITC-DV1 can selectively bind to CXCR4, demonstrating that fluorescence-labeled HAPs can be utilized in competitive binding assays instead of antibody-based competitive analysis with the advantage of cost and efficiency.

### 2.3. Prediction of Ligand Binding Modes Based on FITC-HAP Paired Probes

**Figure 3:**
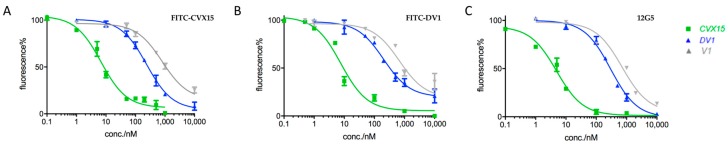
Competitive binding affinities of CXCR4 ligands Type 1 including CVX15, DV1 and V1 respectively in (**A**) FITC-CVX15, (**B**) FITC-DV1, and (**C**) 12G5-based binding assays. Mean ± standard deviation; *n* = 3 independent experiments.

Crystal structures and mutagenesis studies have revealed that FITC-labeled HAPs and antibodies can bind to CXCR4 in different manners: apart from the overlapping region, CVX15 locates into the major pocket; vMIP-II-derived peptides reach into the minor pocket with *N*-terminus; and anti-CXCR4 monoclonal antibody 12G5 occupies the major surface of CXCR4 extracellular regions (but not N-terminus, which is closer to the minor pocket) and interacts with residues Asp182 and Tyr190 [49]. CVX15 is to the best of our knowledge the only peptide ligand that have been validated to bind inside the major pocket of CXCR4 [24]. We analyzed the results of competitive binding capabilities of each molecule from the above three assays. We noticed key differences in their parallel calculated IC_50_ values and categorized them into three types. Molecules including CVX15, V1, and DV1, designated as Type 1, showed comparable IC_50_ values (differences were less than two folds of the minimum values) in three different binding systems (Table 1 and Figure 3).

**Figure 4:**
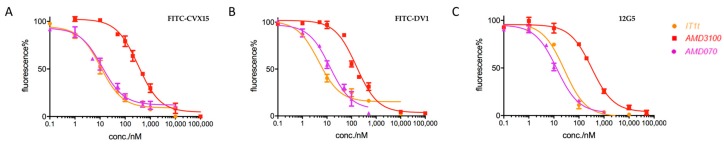
Competitive binding affinities of CXCR4 ligands Type 2 including IT1t, AMD3100 and AMD070 respectively in (**A**) FITC-CVX15, (**B**) FITC-DV1, and (**C**) 12G5-based binding assays. Mean ± standard deviation; *n* = 3 independent experiments.

Interestingly, small molecules including IT1t, AMD070 and AMD3100 with satisfactory binding affinities to CXCR4 revealed significant differences in IC_50_ values achieved from FITC-DV1 competitive assay versus the other two of FITC-CVX15 and 12G5-based assays (Table 1; Figure 4), indicating that these small molecules performed more competitively with FITC-DV1 than with FITC-CVX15. We designated these molecules as Type 2 and concluded that they might locate in the minor pocket of CXCR4 and could therefore compete better with FITC-DV1. This was consistent with evidence from co-crystal structure in the case of CXCR4-IT1t binding complex.

**Figure 5:**
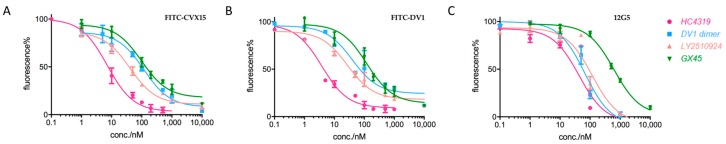
Competitive binding affinities of CXCR4 ligands Type 3 including HC4319, DV1 dimer, LY2510924, and GX45 respectively in (**A**) FITC-CVX15, (**B**) FITC-DV1, and (**C**) 12G5-based binding assays. Mean ± standard deviation; *n* = 3 independent experiments.

Peptides including HC4319, DV1 dimer, and LY2510924, as well as a small organic molecule GX45 discovered in our laboratory were designated as Type 3. They were less competitive in 12G5-based binding assay compared with two HAP-based assays. Their IC_50_ values from 12G5-based assays were over two-fold higher than IC_50_ values from FITC-CVX15 and FITC-DV1 assays (Table 1; Figure 5). We noticed that these molecules are either linear molecules or cyclic peptides with chemical flexibility, unlike rigid molecules such as IT1t and AMD070. We hypothesized that these molecules may bind both into the inner pocket and onto the surface of CXCR4; however, their binding poses on the surface may not be completely consistent with that of antibody 12G5. They may prefer to locate in the minor pocket and *N*-terminus of CXCR4, whereas 12G5 tends to interact with the ECL2 and ECL3 regions. In addition, IT1t and V1 have been validated to locate in the minor pocket by crystal structures and they showed lower affinities in the 12G5-based assay as expected. Our results and analyses were in agreement with binding details revealed by previous epitope mapping studies of 12G5 [49] as well as crystal structures of CXCR4-IT1t, CXCR4-CVX15 [24] and CXCR4-vMIP-II [25].

**Figure 6:**
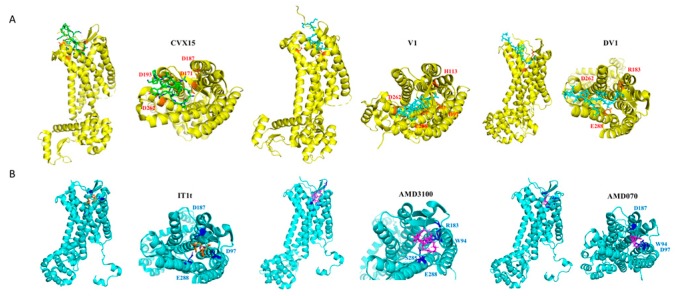
Binding models of diverse CXCR4 ligands from molecular docking or crystallography. (**A**) Binding poses of peptidic inhibitors CVX15, N-terminus of V1 and DV1 by side view and pocket view (presented in green, aquamarine and cyan respectively). (**B**) Binding poses of small-molecule inhibitors IT1t, AMD3100 and AMD070 by side view and pocket view (presented in orange, magenta and purple respectively).

To further validate the above binding mode predictions, we conducted in silico interacting mode analyses and molecular docking studies of several peptides and small molecules (Figure 6). Docking poses of AMD070, AMD3100 and DV1 revealed their interactions with key residues Trp94 and Glu288, providing evidence to support our hypothesis on their preference for minor pocket. Molecular docking studies of our peptidic HC4319 indicated that HC4319 locates preferentially in the minor pocket and interacts with residues Trp94, Asp97 and Glu288 (unpublished data). Previous prediction on the interaction of LY2510924 reported by Lilly Research Laboratories presented key residues Trp94 in the minor pocket and Arg30 on *N*-terminus [50], which was slightly different from epitope mapping result of 12G5. Our hypothesis was also validated by recent CXCR4 mutagenesis studies in our laboratory (unpublished data). Theses combinational results of HAP-based competitive binding systems and molecular modeling studies together provided solid evidence and an effective method for analyzing binding poses and interactive details of diverse CXCR4 ligands.

## 3. Discussion

CXCR4 has long been a classic representative of GPCR family and plays crucial roles in HIV-1 infection and cellular metastasis, making it a target of intense medicinal interest [51]. Structural evidence accumulated recent years has attracted significant attention on the insightful analyses of overlapped and distinguished ligand binding regions on CXCR4. Crystal structures of CXCR4 in complex with ligands proposed a ‘two distinct subpockets’ binding model including a CVX15-based major pocket and an IT1t-based minor pocket. CVX15 is the only ligand proven by crystallography to locate in the major pocket. Our group has developed a series of CXCR4 ligands including both peptides and small molecules and conducted both computational and biological studies in understanding precise and distinctive binding modes of diverse CXCR4 ligands as well as developing novel molecular probes. Though a lot of molecular probes and nanoprobes targeting CXCR4 have been developed recent years, most of them are designed mainly for clinical imaging. Paired peptide probe system was presented in this study with the aim of targeting distinct CXCR4 subpockets. We addressed the peculiarity of FITC-CVX15 and FITC-DV1 in regard for their binding modes in addition to binding affinity and preclinical applications on imaging.

Peptide probes FITC-CVX15 and FITC-DV1 were applied to a cell-based competitive binding system including three parallel binding assays. Results proved both of their abilities for individual binding assays for identifying or screening novel ligands targeting CXCR4. Moreover, together with computational approaches, these parallel results provided a novel method for elucidating interactive patterns between CXCR4 and specific ligands of interest. In consideration of technical difficulties and cost of co-crystallization studies regarding CXCR4 and its ligands, this probe system provided a more readily accessible approach for biochemical profiling on potential ligand binding modes.

In addition, these two peptide probes were highly potent for other biological and clinical applications regarding CXCR4 detection. We applied FITC-CVX15 and FITC-DV1 to analyze CXCR4 expression on overexpressing CHO cell lines by flow cytometry and confocal microscope imaging (Figure S2), and fluorescent signals were both detected in a concentration-dependent manner, indicating their abilities to replace the imaging method involving traditional FITC-conjugated goat anti-mouse IgG and their potential for further development into low-cost efficient clinical imaging tools for CXCR4 expression and distribution analyses.

## 4. Conclusions

In this study, we developed an efficient probe system, including FITC-CVX15 and FITC-DV1, with the aim of targeting distinct CXCR4 subpockets. We conducted rational design of these two probes, characterized their chemical properties, and evaluated their applicability in in vitro biochemical experiments. They were then applied in parallel CXCR4 competitive binding assays together with the FITC-labeled antibody. The parallel assays in combination with computational approaches provided a method for analyzing potential binding modes of diverse CXCR4 ligands based on the ‘two distinct subpockets’ model of CXCR4. In addition, FITC-CVX15 and FITC-DV1 provided alternative tools for flow cytometry analysis and confocal imaging over conventional radioactive or antibody-based probes. In summary, this peptidic fluorescent probe system provided a novel ligand detecting and protein imaging tool, and presented a new approach for analyzing detailed binding affinities and interactive patterns between CXCR4 and various ligands. These probes will aid future discovery and characterization of CXCR4-targeting small molecules with clinical applications for HIV-1 infection, tumor metastasis, and other human diseases mediated by CXCR4.

## 5. Materials and Methods

### 5.1. Chemical Synthesis and Evaluation of FITC-CVX15 and FITC-DV1

Peptide probes FITC-CVX15 and FITC-DV1 were synthesized using semi-automatic multi-channel solid phase peptide synthesizer. FITC-CVX15 was synthesized by coupling aminocaproic acid (Ahx) to residue Lysine 7 and then one FITC group to the Ahx. Synthesis of FITC-DV1 was initiated by coupling sequentially Ahx and whole sequence of DV1 from C-terminal to *N*-terminal to the dde group protected Fmoc-lysine. The dde group was then removed and one FITC group was coupled onto Lysine residue [48]. Chemical properties of FITC-CVX15 and FITC-DV1 probes were evaluated by MALDI-TOF analysis and HPLC respectively.

### 5.2. Determination of Saturation Curves on FITC-CVX15 and FITC-DV1

CXCR4-overexpressing CHO cell line (CHO-CXCR4) was established and cultured according to methods published previously by our group [22]. CHO-CXCR4 cells were selected using G418 sulfate and cultured in DMEM media (HyClone, Logan, UT, USA) supplemented with 10% fetal bovine serum (Gibco, Grand Island, NY, USA) and 100 IU penicillin, 0.1 mg/mL streptomycin. Freshly cultured CHO-CXCR4 were trypsinized and washed with 0.1% bovine serum albumin in phosphate-buffered saline (0.1% BSA/PBS). In saturation binding assays, one hundred microliters of 5 × 10^6^ cells/mL in 1% BSA/PBS were added to each well of a 96-well plate. After centrifugation, 100 μL of FITC-DV1 was added under a gradient of concentrations (10 nM, 50 nM, 100 nM, 200 nM, 400 nM, 800 nM, 1000 nM, and 1200 nM) in 1% BSA/PBS. After sufficient mixing of each well, the plate was covered with an opaque sealing tape and incubated for 50 min on a shaker in a dark environment at 4 °C. The cells were then washed three times and resuspended in 100 μL of 1% BSA/PBS. Fluorescence intensity at λ_excitation_ 485 nm and λ_emission_ 535 nm was determined using a spectrophotometric microplate reader (PerkinElmer EnVision^TM^, Waltham, MA, USA).

### 5.3. 12G5 Antibody-Based Competitive Binding Assay

Gradient concentrations of CXCR4 ligands were prepared with 1% BSA/PBS. CHO-CXCR4 cells were cultured and diluted into 5 × 10^5^ cells/mL in 0.5% BSA/PBS. One hundred microliters of above cell solutions were added to each well of a 96-well plate and washed twice with 0.5% BSA/PBS. Cells were then incubated with 100 μL primary 12G5 Ab (1:2000, mouse anti-human CD184 antibody, BD Biosciences, CA, USA) in 0.5% BSA/PBS except for negative control group and gradient concentrations of CXCR4 ligands for 40 min on a shaker at 4 °C. Cells were then washed twice with 0.5% BSA/PBS and incubated with a solution of 50 μL secondary antibody in 0.5% BSA/PBS (1:200, FITC conjugated goat anti-mouse IgG, Sigma-Aldrich, MO, USA) for 30 min on a shaker in a dark environment at 4 °C and then washed twice with 0.5% BSA/PBS. Fluorescence intensity at λ_excitation_ 485 nm and λ_emission_ 535 nm was determined using a spectrophotometric microplate reader (PerkinElmer EnVision^TM^, Waltham, MA, USA).

### 5.4. FITC-CVX15/FITC-DV1-Based Competitive Binding Assays

Similarly, CHO-CXCR4 cells were added at 5 × 10^5^ cells/100 μL per well to a 96-well plate and incubated in 1% BSA/PBS for 20 min. After centrifugation, 100 μL of 400 nM FITC-CVX15 or 1 μM FITC-DV1 in 1% BSA/PBS were added to each well except for negative control group. Gradient concentrations of CXCR4 ligands were then added to each corresponding well. After sufficient mixing of each well, the plate was covered with an opaque sealing tape and incubated for 50 min on a shaker in a dark environment at 4 °C. Cells were then washed twice by 100 μL of 1% BSA/PBS. Fluorescence intensity at λ_excitation_ 485 nm and λ_emission_ 535 nm was determined using a spectrophotometric microplate reader (PerkinElmer EnVision^TM^, Waltham, MA, USA).

### 5.5. Flow Cytometry Analysis on Binding of Peptide Probes

Freshly cultured CHO-CXCR4 cells were trypsinized and washed as described above and incubated for 50 min with FITC-CVX15 and FITC-DV1 probes in solution at a gradient concentration of 1 nM, 10 nM, 50 nM, 100 nM and 500 nM on a shaker in a dark environment at 4 °C. After incubation, cells were washed for three times with 0.1% BSA/PBS. Within 10 min before flow cytometry analysis, propidium iodide was added to distinguish live and dead cells. Cells incubated without peptides served as controls.

### 5.6. Imaging Analysis by Confocal Laser Scanning Microscope

CHO and CHO-CXCR4 cells were cultured in 35-mm dishes for 16 h before imaging analysis. Adherent cells were incubated with FITC-CVX15, FITC-DV1 probes, and controls of CVX15, DV1, and FITC solutions for 30 min at room temperature. In antibody groups, cells were incubated with antibody 12G5 for 40 min followed by FITC-conjugated goat anti-mouse IgG for 30 min at room temperature. After incubation, cells were washed for three times with 1% BSA/PBS. Imaging analysis was performed using a laser scanning confocal microscope LSM710META (Zeiss, Germany) at λ_excitation_ 485 nm.

### 5.7. Hardware and Software

In silico studies were performed on Dell Workstation Precision 5810 with 2E5-2600 V3 2.133 GHz processor, 6 GB RAM, and 256 GB hard drive running in Red Hat Enterprise Linux (RHEL) operating system. Bioinformatics software SYBYL-X 2.1.1 (Tripos Associates, St. Louis, MO, USA) was used for molecular docking simulations. Chimera (UCSF, CA, USA) and PyMol Molecular Graphics software (Schrödinger, NY, USA) helped analyze details of protein–ligand interactions. Online resources including protein data bank (http://www.rcsb.org/pdb/) were utilized to perform this study.

### 5.8. Molecular Docking Studies of CXCR4-Ligand Interactions In Silico

Molecular modeling studies of ligand-CXCR4 complexes were carried out using SYBYL-X 2.1.1 software according to procedures previously developed in our laboratory [52]. Surflex-Dock (SFXC) algorithm serves as an automatic molecular docking program using an empirical scoring function and a patented searching engine [53]. A negative-phase “protomol” with a threshold of 0.50 and bloat set to 0 Å was generated for each protein structure. During docking process, the maximum number of poses per ligand was set to 20. Other parameters were established using default values. After docking, minimized ligand poses and their rankings were returned. Consensus scores (C_Scores), representing an overall of Dock_Scores, PMF_Scores, Gold_Scores, and Chem_Scores were provided for ranking the binding affinity of ligands to the receptor (SYBYL C_Score module = Dock_Score + PMF_Score + Gold_Score + Chem_Score). Crash represented the degree of inappropriate penetration into the protein as well as self-clashing of ligands. Amino acid interactions inside each complex structure were analyzed by Chimera and PyMol.

### 5.9. Statistical Analysis

Biological assays were performed in triplicate and data were analyzed in Microsoft Excel and plotted in GraphPad Prism 7 (GraphPad Software Inc., La Jolla CA, USA). Average values were expressed as mean ± SD, n ≥ 3 independent experiments.

## Figures and Tables

**Table 1:** Summary on biological affinities (presented by IC_50_ values) of diverse CXCR4 ligands in FITC-CVX15, FITC-DV1, and 12G5-based competitive binding assays.

Category	Name\IC_50_ (nM)	FITC-CVX15	FITC-DV1	12G5 Ab
Type 1	CVX15	6.2	6.8	6.0
	V1	687.4	676.5	712.5
	DV1	238.2	203.6	296.9
Type 2	IT1t	19.3	6.0	26.2
	AMD3100	314.5	104.9	324.3
	AMD070	14.7	5.0	12.2
Type 3	HC4319	7.1	5.0	46.6
	DV1 dimer	29.4	25.6	67.0
	LY2510924	35.4	33.9	117.4
	GX45	137.4	139.1	628.1

## Supplementary Materials

The supplementary materials are available online.

## Abbreviations

CXCR4	CXC-type chemokine receptor 4
HIV-1	Human immunodeficiency virus type 1
GPCR	G protein-coupled receptor
JAK	Janus kinase
STAT	Signal transducer, and activator of transcription
PPIs	Protein-protein interactions
HAPs	High-affinity peptides
PE	Phycoerythrin
MALDI-TOF	Matrix-assisted laser desorption ionization time-of-flight
HPLC	High Performance Liquid Chromatography
FCM	Flow cytometry
Ahx	Aminocaproic acid
BSA	Bovine serum albumin
PBS	Phosphate-buffered saline
SFXC	Surflex-Dock algorithm
