# Supplementary material for: Structural and Biological Characterizations of Novel High-Affinity Fluorescent Probes with Overlapped and Distinctive Binding Regions on CXCR4
Source: Molecules. 2019 Aug 13;24(16):2928. doi: 10.3390/molecules24162928 (PMC6720714; doi:10.3390/molecules24162928)
Supplement: Supplementary file 1 [file molecules-24-02928-s001.pdf]

molecules

Supplementary information

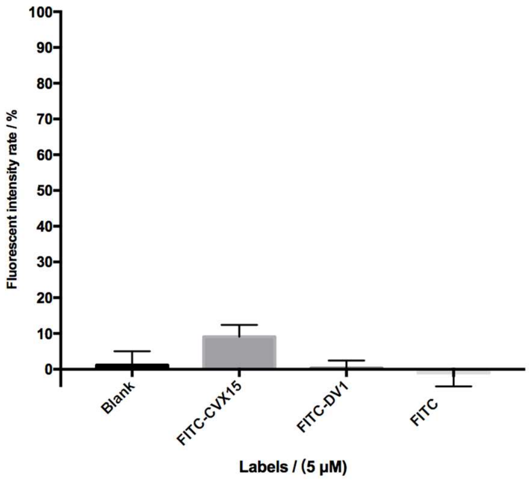

Figure S1. Fluorescence intensity percentages of FITC-CVX15, FITC-DV1 and FITC labels at a concentration of 5 μM on CHO-CCR5 cells with CCR5 overexpression versus CHO cells without CCR5 overexpression. No fluorescent label (blank) and FITC only groups served as controls. FITC-CVX15 and FITC-DV1 did not show strong binding to CCR5 which is consistent with their CXCR4 receptor selectivity.

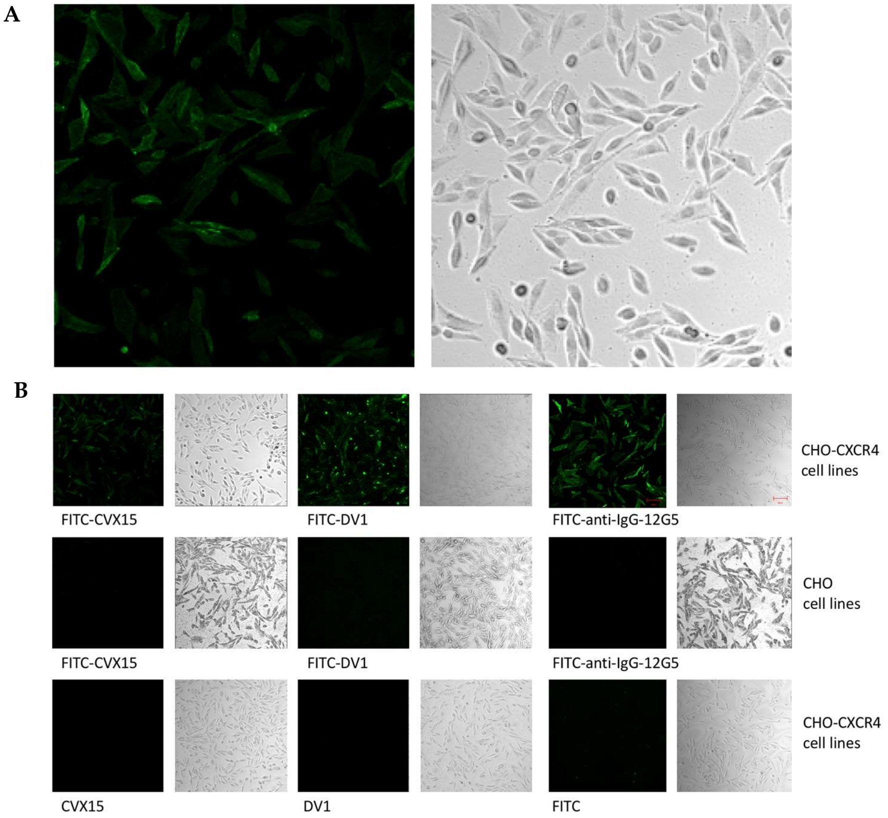

Figure S2. (**A**) Confocal imaging results of FITC-CVX15 on CHO-CXCR4 cell lines. FITC fluorescence was detected at an excitation wavelength of 488 nm (left) and observation of cellular morphology

and distribution was presented accordingly under bright field illumination (right). (**B**) Summary of confocal images of CHO-CXCR4 and CHO cell lines stained with FITC-CVX15, FITC-DV1 and FITC-anti-IgG-12G5 respectively at an excitation wavelength of 488 nm and under bright field illumination.
